# The Ratio of C-Reactive Protein to Albumin Is an Independent Predictor of Malignant Intraductal Papillary Mucinous Neoplasms of the Pancreas

**DOI:** 10.3390/jcm10102058

**Published:** 2021-05-11

**Authors:** Simone Serafini, Alberto Friziero, Cosimo Sperti, Lorenzo Vallese, Andrea Grego, Alfredo Piangerelli, Amanda Belluzzi, Lucia Moletta

**Affiliations:** 1 Department of Surgery, Oncology and Gastroenterology, 3rd Surgery Clinic, University of Padua, via Giustiniani 2, 35128 Padua, Italy; simone.serafini@ymail.com (S.S.); alberto.friziero@aopd.veneto.it (A.F.); d.andrea.grego@gmail.com (A.G.); alfredopiange@gmail.com (A.P.); amandabelluzzi@gmail.com (A.B.); lucia.moletta@unipd.it (L.M.); 2 Department of Surgery, SS. Giovanni and Paolo Hospital, Sestiere Castello 6777, 30122 Venice, Italy; l.vallese@gmail.com

**Keywords:** biomarker, C-reactive protein to albumin ratio, inflammation, intraductal papillary mucinous neoplasm, modified Glasgow prognostic score, neutrophyl lymphocite ratio, pancreatic cancer, platelet-to-lymphocyte ratio

## Abstract

There is growing evidence to indicate that inflammatory reactions are involved in cancer progression. The aim of this study is to assess the significance of systemic inflammatory biomarkers, such as the neutrophil-to-lymphocyte ratio (NLR), the platelet-to-lymphocyte ratio (PLR), the ratio of C-reactive protein to albumin ratio (CAR), the prognostic nutritional index (PNI) and the modified Glasgow prognostic score (mGps) in the diagnosis and prognosis of malignant intraductal papillary mucinous neoplasms (IPMNs) of the pancreas. Data were obtained from a retrospective analysis of patients who underwent pancreatic resection for IPMNs from January 2005 to December 2015. Univariate and multivariate analyses were performed, considering preoperative inflammatory biomarkers, clinicopathological variables, and imaging features. Eighty-three patients with histologically proven IPMNs of the pancreas were included in the study, 37 cases of low-grade or intermediate dysplasia and 46 cases of high-grade dysplasia (HGD) or invasive carcinoma. Univariate analysis showed that obstructive jaundice (*p* = 0.02) and a CAR of >0.083 (*p* = 0.001) were predictors of malignancy. On multivariate analysis, only the CAR was a statistically significant independent predictor of HGD or invasive carcinoma in pancreatic IPMNs, identifying a subgroup of patients with a poor prognosis. Combining the CAR with patients’ imaging findings, clinical features and tumor markers can be useful in the clinical management of IPMNs. Their value should be tested in prospective studies.

## 1. Introduction

In the pancreas, intraductal papillary mucinous neoplasms (IPMNs) originate from the mucinous epithelium of the pancreatic ductal system. Their incidence is rising, probably due to an increasingly extensive use of cross-sectional imaging, but their management remains controversial. According to the World Health Organization (2010), IPMNs are premalignant lesions showing a broad spectrum of dysplastic changes. According to the different involvement of duct system, IPMNs are divided into main duct type (MD-IPMN), branch duct type (BD-IPMN), and mixed type (MT-IPMN). For the purposes of pathological grading, IPMNs are classified as low-, intermediate- or high-grade dysplasia, or invasive carcinoma. IPMNs with low- and intermediate-grade dysplasia are defined as benign, while the malignant IPMNs include those classified as high-grade dysplasia (HGD) or invasive carcinoma [1]. Benign IPMNs can potentially be managed conservatively, whereas malignant IPMNs require surgical resection in accordance with international guidelines. Malignant IPMNs carry a worse prognosis than benign IPMNs, making it essential to predict the malignant potential of an IPMN accurately at the time of its diagnosis or follow-up. Several clinical and radiological parameters have been considered over time with a view to stratifying the malignant potential of pancreatic IPMNs and thereby facilitate their management. International consensus guidelines (ICG) recommend surgery for cases with one or more “high-risk stigmata” (HRS), while further assessment with endoscopic ultrasonography is suggested for cases with “worrisome features” (WF) [2]. The accuracy of these guidelines in detecting early invasive carcinoma in IPMNs is limited, however [3]. Even conventional tumor markers like CEA and CA 19.9 are not very useful in predicting the risk of malignancy in this setting [4]. There is still a crucial need for markers capable of identifying which IPMNs warrant surgical treatment.

There is growing evidence to indicate that inflammatory reactions and nutritional status are involved in cancer progression. Many host-related inflammatory biomarkers measurable in peripheral blood samples have been investigated as potentially effective prognostic factors in several types of cancer [5,6,7], including pancreatic adenocarcinoma [8,9]. These serum parameters include, among others, the neutrophil-to-lymphocyte ratio (NLR), the platelet-to-lymphocyte ratio (PLR), the ratio of C-reactive protein to albumin (CAR), the prognostic nutritional index (PNI) and the modified Glasgow prognostic score (mGps). The prognostic impact of these markers in patients with IPMNs has yet to be well established, and the literature reports different results.

The NLR alone was described as an independent predictor of IPMN-associated invasive carcinoma, but its sensitivity was not high enough to distinguish between degrees of dysplasia in IPMNs [10]. On the other hand, a significant prognostic indication of malignancy (both HGD and invasive carcinoma) in IPMNs could only be reached by combining the preoperative NLR and PLR with tumor markers and imaging findings [11,12]. In short, there is still not enough evidence regarding the predictive role of these biomarkers.

Herein we present a retrospective study on a series of patients whose IPMNs were resected in an effort to establish the value of the NLR, PLR, CAR, PNI and mGps in predicting which cases of IPMN are malignant.

## 2. Materials and Methods

Between January 2005 and December 2015, all consecutive patients with pancreatic IPMNs who underwent surgical resection at our Department were identified using a prospectively maintained database. The data were analyzed retrospectively. The conventional workup included blood tests, tumoral markers, contrast-enhanced magnetic resonance imaging (MRI), bilio-pancreatic endoscopic ultrasound (EUS) and positron emission tomography (PET) with fluorine-18-fluorodeoxyglucose if clinically appropriated. Exclusion criteria were adopted to avoid potential confounding factors, including: a history of other malignancies, autoimmune disease or transplantation requiring immunosuppressant and steroid therapies; cholangitis or other forms of infection; pancreatic endocrine tumors and cystic neoplasms other than IPMNs. Patients diagnosed with metastases were not included. Disease-related symptoms such as visceral abdominal pain, dyspepsia (upper abdominal fullness, nausea, belching) and compression syndrome (experience of feeling full earlier than expected when eating, vomiting) were collected. IPMNs were confirmed on pathological examination of surgical specimens in all cases. The cases were all retrieved from the archives of the Surgical Pathology and Cytopathology Unit at Padova University Hospital (Padova, Italy). Surgical specimens were examined to histologically classify cases as low-, intermediate or HGD or invasive carcinoma, based on the recommendations of the Baltimore consensus [13]. IPMNs were described as main duct (MD), branch duct (BD) or combined type, based on preoperative imaging. Main-duct IPMNs are characterized by involvement of the main pancreatic duct, with or without associated involvement of the branch ducts (in the former case, they are called combined-type IPMNs). They usually present as a dilated (≥1 cm) main pancreatic duct, or as a cystic dilation of the main duct and its branches. Branch-duct IPMNs originate in the side branches of the pancreatic ductal system, appearing as cystic lesions communicating with a main pancreatic duct showing no dilations [14]. All resection procedures were performed by the same surgical team, in accordance with the International Consensus Guidelines of the time [2]. Surgery involved pylorus-preserving pancreaticoduodenectomy (PD) for tumors of the head of the pancreas, or distal pancreatectomy with or without splenectomy, for tumors of the body and tail. Total pancreatectomy was reserved for cases where the resection margins of the pancreas were affected by the tumor. More parenchyma-sparing resections of the pancreas, such as central pancreatectomy and tumor enucleation, were performed in the case of small to medium-sizes lesions localized in the pancreatic body.

Based on the pathological grading of the resected IPMNs, patients were divided into a benign group (IPMNs with low- and intermediate-grade dysplasia) and a malignant group (IPMNs associated with HGD and invasive carcinoma) [1]. Each patient’s clinical and pathological records were reviewed, and the following characteristics were included in our analysis: gender; age; abdominal pain; dyspepsia; tumor site; compression syndrome; tumor size; main pancreatic duct (MPD) diameter; cyst diameter; HRS; WF; CEA; CA 19.9; and inflammatory biomarkers, such as the NLR, PLR, CAR, PNI and mGps. Preoperative serum levels of CA 19-9 (RIA, Centocor Inc., Malvern, PA, USA, reference: <37 kU/L) and CEA (EIA Kit, General Biologicals Inc., Taiwan, reference: <5 ng/mL) were recorded. Neutrophil, lymphocyte and platelet counts, and serum albumin levels (g/dL) were obtained from the latest blood sample collected just before surgery. The following indexes were retrospectively calculated: the NLR (the ratio of the absolute neutrophil count to the absolute lymphocyte count in the blood cell count); the PLR (the ratio of the absolute platelet count to the absolute lymphocyte count in the blood cell count); the CAR (the ratio of C-reactive protein [CRP] (mg/dL) to albumin (g/dL); the PNI (calculated as 10 × serum albumin + 0.005 x total lymphocyte counts) and the mGps, which ranged from 0 to 2. Patients with both high CRP levels (>10 mg/L) and hypoalbuminemia (<35 g/L) had a mGps of 2; those with normal CRP and albumin levels scored 0; and those with high CRP levels and normal albumin levels scored 1, in line with recent data [15].

Ethical approval: All procedures performed in studies involving human participants were in accordance with the ethical standards of the institutional and/or national research committee and with the 1964 Helsinki Declaration and its later amendments or comparable ethical standards.

Statistical analyses were run using STATA, version 14.1 (4905 Lakeway Drive College Station, Midtown Dr, TX, 77845, USA). Continuous and categorical variables are reported as medians with the interquartile range (IQR), and as whole numbers (percentages), respectively. The diagnostic accuracy, including sensitivity and specificity, was calculated for each host-derived inflammatory biomarker using the cutoffs obtained from receiver operating characteristic (ROC) curve analysis. The optimal cutoff was identified as the point of intersection nearest the top left-hand corner between the ROC curve and a diagonal line drawn from the top right-hand corner to the bottom left-hand corner of the graph. The sensitivity, specificity, positive predictive value (PPV), negative predictive value (NPV), and accuracy of the inflammatory biomarkers and radiological imaging findings in differentiating between malignant and benign IPMNs were calculated according to the following formulas: sensitivity = true positive (TP)/[TP + false negative (FN)]; specificity = true negative (TN)/[TN + false positive (FP)]; PPV = TP/[TP + FP ]; NPV = TN/[TN + FN] and accuracy = [TP + TN]/[TP + TN + FP + FN]. For the univariate analysis, patients were divided into the two groups, with benign and malignant IPMNs. Differences between the characteristics of the patients in these two groups were tested for significance using the Mann-Whitney U test and chi-square test, as appropriate. A multivariate analysis was performed using the logistic regression model, and including significant variables identified in our univariate analysis only. The effect size of the odds ratio (OR) is presented with the 95% confidence interval (CI). Box plot graphs were drawn to represent the distribution of the inflammatory biomarkers of proven prognostic significance.

## 3. Results

Table 1 shows the clinicopathological features of the whole study cohort, comprising 83 patients with proven IPMNs. The sample was a median 69 years old (range 43–86), and consisted of 45 males and 38 females. No cases of concomitant pancreatic ductal adenocarcinoma were detected. None of the patients received neoadjuvant therapies. Among the 83 patients, 40 patients (48%) had main-duct IPMNs, 10 (12%) had branch-duct IPMNs, and 33 (40%) had combined-type IPMNs. Thirty-seven tumors (44.5%) were benign (30 with low-grade dysplasia, and 7 with intermediate-grade dysplasia), and 46 (55.5%) were malignant (7 with HGD, and 39 with invasive carcinoma). The surgical procedure involved pylorus-preserving PD in 50 patients, distal pancreatectomy with splenectomy in 22, distal pancreatectomy without splenectomy in 7, and total pancreatectomy in one. Tumor enucleation and central pancreatectomy were performed in two and one patient, respectively.

**Figure 1:**
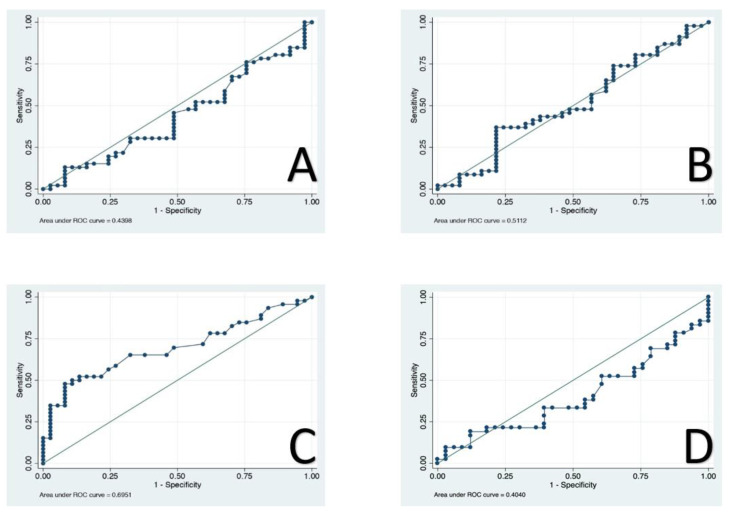
Receiver operator characteristic (ROC) curves of neutrophil-to-lymphocyte ratio (NLR) (**A**), platelet-to-lymphocyte ratio (PLR) (**B**), C-reactive protein to albumin ratio (CAR) (**C**), prognostic nutritional index (PNI) (**D**).

Four inflammatory biomarkers were examined and ROC curve analysis was set to identify the optimal cutoffs for the NLR, PLR and CAR, while the mGps was calculated for each patient. The optimal cutoffs were: 2.38 for the NLR (AUC 0.43, sensitivity of 46% and specificity of 51%); 185.5 for the PLR (AUC 0.51, sensitivity of 37% and specificity of 78%); 0.083 for the CAR (AUC 0.69, sensitivity of 60% and specificity of 70%); and 42.05 for the PNI (AUC 0.46, sensitivity of 52% and specificity of 39%) (Figure 1).

Univariate and multivariate analyses were performed to examine the causal relationship between the presence of IPMNs with a malignant potential and the inflammatory biomarkers (Table 2), inputting patients’ preoperative clinicopathological variables and imaging features (Table 3).

On univariate analysis obstructive jaundice (*p* value 0.022) and a CAR of >0.083 (*p* value 0.001) emerged as predictors of malignancy. On multivariate analysis, only the CAR was an independent predictor of malignant IPMN (OR 7.9, IQR 2.01–31.83, *p* 0.003). No significant differences in gender, age, preoperative abdominal pain, dyspepsia or compression syndrome came to light between patients with benign as opposed to malignant IPMNs. Nor did the two groups differ in terms of tumor size, tumor site, MPD diameter or cyst diameter. The median serum levels of tumor markers CA 19.9 and CEA were not significantly different in the two groups of patients with IPMN. Among the preoperative risk-related parameters considered, the frequency of enhancing mural nodules, MPD ≥ 10 mm, cyst ≥ 3 cm, enhancing cyst walls, pancreatitis, MPD 5–10 mm, and abrupt MPD caliber changes with distal pancreatic atrophy did not differ between the malignant and benign IPMN groups (*p* > 0.05 for all).

**Figure 2:**
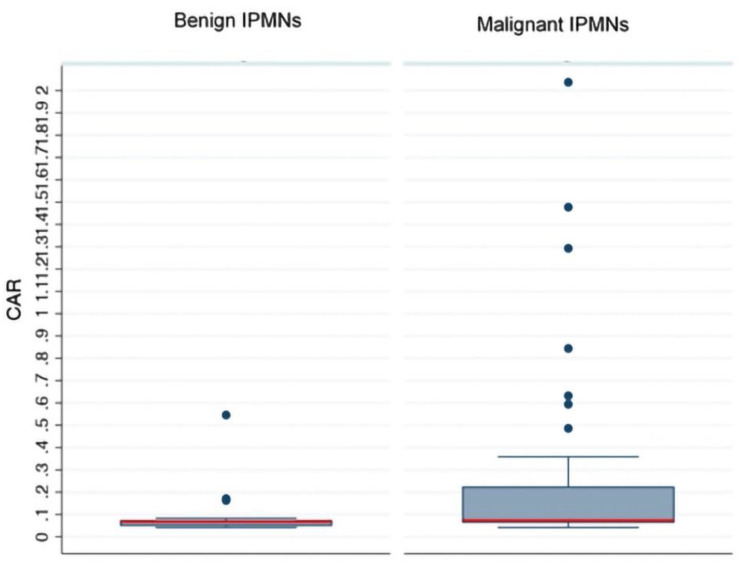
Quantification of CAR values in benign and malignant intraductal papillary mucinous neopla (IPMNs). The red horizontal bar represents the optimal cut off (0.083) obtained with receiver operator characteristic curves analysis and the blue ones represent the 25th and 75th percentiles. CAR: C-reactive protein to albumin ratio.

The distribution of the CARs in the two groups is represented with a box plot (Figure 2). Most of the patients with benign IPMNs had a CAR lower than the study cutoff, while almost all those with malignant IPMNs had a CAR higher than 0.083.

The sensitivity, specificity, PPV, NPV and accuracy of the CAR in detecting malignancy were 52%, 93%, 91%, 50%, and 66%, respectively, vs. 74%, 50%, 72%, 54%, and 66%, respectively, for the ICG criteria for HRS. Combining the two parameters (patients who demonstrate HRS according to ICG criteria and simultaneously CAR values > 0.083) resulted in a 43% sensitivity, 97% specificity, 94% PPV, 55% NPV, and 63% accuracy for the diagnosis of malignancy.

**Figure 3:**
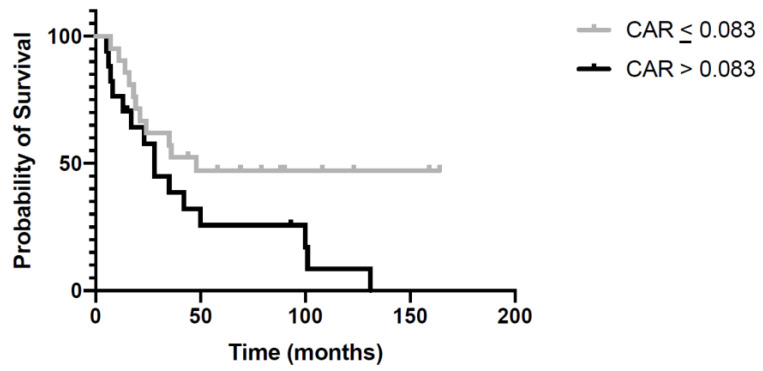
Kaplan–Meier curve for overall survival estimated for patients with C-reactive protein to albumin ratio (CAR) values > 0.083 or ≤ 0.083. CAR > 0.083 was significantly associated with worse survival (*p* = 0.004).

To judge the prognostic impact of the CAR, we first analyzed that there are no statistically significant differences between the clinicopathological and inflammatory parameters in the patients with high and low CARs, then we examined the association between high CARs and long-term outcomes in the 39 cases of IPMN with invasive carcinoma. Figure 3 shows the survival curves for patients with a high vs. low CAR: those with a higher CAR had a significantly shorter overall survival (*p* = 0.004) than those with a lower CAR.

## 4. Discussion

The present study suggests that the CAR is useful for predicting HGD and invasive carcinoma in patients with IPMNs, and that the value of the CAR in detecting malignancies is independent of the well-established parameters indicated in the international guidelines [2,14,16]. Univariate analysis found obstructive jaundice, and a CAR >0.083 significantly associated with malignant IPMNs, but multivariate analysis showed that only the CAR was an independent predictor of malignant lesions (both HGD and invasive carcinoma). Although the sensitivity of the CAR in detecting malignancies was low, its specificity was higher than that of the ICG criteria for malignant IPMNs. Combining the ICG criteria with the CAR achieved only a slight increase in the specificity and PPV.

The CAR is easily obtained from a simple blood test. It is inexpensive and can be calculated by clinicians both at the initial examination and during the follow-up of patients with IPMNs. Although the CRP preoperative assessment is not a common practice, in our institution, it is included in protocols for research purposes for pancreatic diseases. However, it is becoming more and more common in the standard workup for elective surgery during COVID-19 era. To the best of our knowledge, this is the first study to emphasize the role of the CAR in identifying the malignant potential of IPMNs.

Features previously suggested to predict malignancy (HGD or invasive carcinoma) in IPMNs include: an association with symptoms; cyst wall thickening; mural nodules; MPD dilation; abrupt pancreatic duct caliber changes; lymphadenopathy; and higher than normal CA19-9 or CEA serum levels [17]. The rate of pointless surgical procedures for overestimated pancreatic lesions remains high, however, which is why new, specific biomarkers are needed for a better clinical management of patients with IPMNs. Both the NLR and the PLR have recently been proposed as predictors of an invasive carcinoma in pancreatic cysts [18,19]. High NLRs and PLRs may be due to neutrophilia and a systemic inflammatory response during the development of invasive cancer [20,21], and to a declining lymphocyte counts caused by immune system suppression [22]. The value of the preoperative NLR in predicting the malignant potential of IPMNs has been emphasized by several authors [23,24,25], but the picture remains unclear. While Hata et al. [26] found the preoperative NLR able to predict cases of IPMN with HGD, it was not helpful in differentiating between high-grade and low-grade lesions in the study by McIntyre et al. [24].

In a series of 318 patients with pancreatic cystic neoplasms (including 86 IPMNs), Goh et al. [27] found that a high preoperative PLR, but not the NLR, was an independent predictor of malignancy. They also found that adding the PLR to the ICG criteria improved the accuracy of the latter in detecting cases of invasive carcinoma.

A systemic inflammatory response has increasingly been recognized as an important factor in the process of carcinogenesis and a cancer’s subsequent behavior, with tumor growth appearing to be directly proportional to the degree of inflammation [28,29]. The NLR [23,30,31] or PLR [27], or both [32] have been suggested as useful prognostic markers in patients with pancreatic cancer. A systematic review and meta-analysis conducted by Zhou et al. [33] on 8252 patients with pancreatic cancer showed that a low NLR was significantly associated with better disease-free and overall survival rates than a high NLR. Patients with a low NLR had significantly smaller tumors, better differentiation, earlier-stage disease and low CA 19-9 levels.

The CAR had been previously investigated in pancreatic cancer patients. Haruki et al. [34] found it an independent and significant indicator of poor long-term outcomes after pancreatic resection. Liu et al. [35] also reported finding that a high CAR was an independent factor pointing to a poor prognosis in pancreatic cancer patients. The prognostic implications of inflammatory biomarkers in different types of cancer have also already been reported [36,37].

In our study, the CAR was a predictor of long-term survival in patients with IPMNs associated with invasive carcinoma: patients with a lower CAR had a significantly better survival than those with CAR > 0.083 (*p* = 0.004). This would indicate that the CAR is an important prognostic parameter to consider in the clinical management of patients with IPMNs.

Furthermore, CAR is a new predictive indicator that reflects both inflammatory and nutritional status of cancer patients. This aspects play an important role in carcinogenesis and tumor progression. The real mechanism of the relationship between CAR and survival is unclear. Elevated CRP levels reflect an inflammatory response to tumor necrosis or local tissue damage, which are both factors that condition the stromal microenvironment for the engraftment and growth of metastases [38]. Moreover, PCR has been associated with inhibiting mechanisms of tumor cell apoptosis and an increased production of endothelial growth factors [39]. Likewise, hypoalbuminemia is often observed in patients with malignant diseases and is usually correlated with malnutrition and cachexia, aspects that inevitably affect the poor prognosis of these patients [40,41].

Our study has some limitations that need to be mentioned. First, the design was retrospective, so the possibility of selection bias exists. Second, the sample was small and studies on larger samples will be needed before any definitive conclusions can be drawn. Third, we did not investigate patients whose IPMNs had not been histologically confirmed, or patients who underwent surveillance alone: these patients could be included in future prospective studies. These partial results will have to be validated in further international, randomized multicentric trials in order to increase the number of enrolled patients, reduce selection bias and sample heterogeneity.

## 5. Conclusions

The preoperative CAR is an independent predictor of HGD or invasive carcinoma in IPMNs and identifies a subgroup of patients with a poor prognosis. Combined with imaging findings, clinical features and tumor markers, the CAR can be useful in the clinical management of IPMNs, and its value should be further investigated in international multicentric randomized clinical studies.

## Figures and Tables

**Table 1:** Clinicopathological features of the whole cohort. IPMN, intraductal papillary mucinous neoplasm; MD, main duct; BD, branch duct; MPD, main pancreatic duct.

Variables	Whole Cohort (*n* = 83)
Sex, male *n*, %	45 (54%)
Female *n*,%	38 (46%)
Age, median (IQR range), y	69 (62–76)
IPMN type, *n* (%)	
MD_IPMNs	40 (48%)
BD_IPMNs	10 (12%)
Combined type_IPMNs	33 (40%)
Surgical procedure *n* (%)	
Pancreaticoduodenectomy	50 (60%)
Distal pancreatectomy + splenectomy	22 (27%)
Spleen-preserving distal pancreatectomy	7 (9%)
Central pancreatectomy	1 (1%)
Total pancreatectomy	1 (1%)
Tumor enucleation	2 (2%)
Histological grade, *n* (%)	
Low-grade dysplasia	30 (36%)
Intermediate dysplasia	7 (8%)
High-grade dysplasia	7 (8%)
Invasive carcinoma	39 (47%)
High-risk stigmata *n* (%)	
Obstructive jaundice	18 (22%)
Enhancing solid component	47 (57%)
MPD ≥ 10 mm	12 (14%)
Worrisome features *n* (%)	
Tumor ≥ 3 cm	26 (31%)
Pancreatitis	22 (27%)
Enhancing cyst wall	23 (28%)
MPD 5–9 mm	38 (46%)
Abrupt change in caliber of pancreatic duct with distal pancreatic atrophy	39 (47%)

**Table 2:** Clinicopathological features and biomarkers univariate and multivariate analysis for predicting malignant intraductal papillary mucinous neoplasm (IPMNs). Statistically significant values are in bold. OR: odds ratio; CI: confidence interval; NC: not calculated; IQR: interquartile range; MPD: main pancreatic duct; mGps: modified Glasgow prognostic score; NLR: neutrophil-to-lymphocyte ratio; PLR: platelet-to-lymphocyte ratio; CAR: C-reactive protein to albumin ratio; PNI: prognostic nutritional index.

			Univariate	Multivariate	
Variables	Benign IPMNs (*n* = 37)	Malignant IPMNs (*n* = 46)	*p* Value	OR (95% CI)	*p* Value
Sex, male *n* (%)	22 (59.46%)	23 (50%)	0.39	NC	
Age, median (IQR)	67.5 (62–73.5)	71 (62–78)	0.18	NC	
Abdominal pain *n* (%)	21 (56.76%)	18 (39.13%)	0.12	NC	
Dyspepsia *n* (%)	21 (56.76%)	17 (36.96%)	0.181	NC	
Cephalic location *n* (%)	27 (72.97%)	27 (58.70%)	0.175	NC	
Compression syndrome *n* (%)	13 (35.14%)	7 (15.22%)	0.093	NC	
Tumor size cm, median (IQR)	2 (1.8–3)	2.8 (2–3.2)	0.084	NC	
MPD diameter mm, median (IQR)	6 (3–8)	7 (4–8)	0.14	NC	
Cyst diameter cm, median (IQR)	3 (2–4)	3 (2–4)	0.69	NC	
High-risk stigmata, *n* (%)					
Obstructive jaundice	13 (35.14%)	5 (10.87%)	**0.022**	0.37 (0.08–0.158)	0.18
Enhancing mural nodule	25 (67.57%)	22 (47.83%)	0.09	NC	
MPD > 10 mm	7 (18.92%)	5 (10.87%)	0.54	NC	
Worrisome features, *n* (%)					
Cyst size > 3 cm	12 (32.43%)	14 (30.43%)	0.97	NC	
Pancreatitis	13 (35.14%)	9 (19.57%)	0.27	NC	
Enhancing cyst wall	10 (27.03%)	13 (28.26%)	0.71	NC	
MPD 5–10 mm	17 (45.95%)	21 (45.65%)	0.96	NC	
Abrupt change in caliber of pancreatic duct with distal pancreatic atrophy	17 (45.95%)	22 (47.83%)	0.39	NC	
CA19.9, median (IQR)	4 (2–11)	24 (4–253)	0.42	NC	
CEA, median (IQR)	1 (0–3)	2 (1–5)	0.17	NC	
mGps				NC	
0	31 (83.78%)	37 (80.43%)	0.46		
1	4 (10.81%)	4 (8.70%)	0.52		
2	2 (5.41%)	5 (10.87%)	0.32		
NLR				NC	
<2.38	17 (45.95%)	24 (52.17%)	0.573		
>2.38	20 (54.05%)	22 (47.83%)			
PLR				NC	
<185.5 *n* %	28 (75.68%)	29 (63.04%)	0.217		
≥185.5 *n* %	9 (24.32%)	17 (36.96%)			
CAR					
<0.083 *n* %	31 (83.78%)	22 (47.83%)	**0.001**	7.9 (2.01–31.83)	**0.003**
≥0.083 *n* %	6 (16.22%)	24 (52.17%)			
PNI				NC	
<42.05 *n* %	14 (37.84%)	26 (56.52%)	0.15		
>42.05 *n* %	23 (62.16%)	20 (43.48%)	0.07		

**Table 3:** Distribution of clinicopathological features and biomarkers between patients with high and low CAR. CAR: C-reactive protein to albumin ratio; IQR: interquartile range; MPD: main pancreatic duct; CRP: C reactive protein; PNI: prognostic nutritional index; NLR: neutrophil-to-lymphocyte ratio; PLR: platelet-to-lymphocyte ratio.

Variables	CAR ≤ 0.083 (*n* 53)	CAR ≥ 0.083 (*n* 30)	*p* Value
Sex, male *n*, %	28 (52.83%)	17 (56.67)	0.46
Age, median (IQR)	69.5 (63–76.5)	68 (61–75)	0.42
Abdominal pain *n*, %	27 (50.94%)	12 (41.38%)	0.28
Dyspepsia *n*, %	28 (52.83%)	10 (35.71%)	0.11
Cephalic location *n*, %	36 (67.92%)	18 (60%)	0.31
Compression syndrome *n*, %	13 (25%)	7 (25.93%)	0.57
Tumour size cm, median (IQR)	3 (2–3.75)	2.5 (1.8–3.3)	0.06
MPD diameter mm, median (IQR)	6 (4–9)	5.5 (3.5–7.5)	0.12
Cyst diameter cm, median (IQR)	3 (2–4)	2.6 (2–3.8)	0.26
High risk stigmata, *n* (%)			
Obstructive jaundice	12 (22.64%)	6 (20%)	0.5
Enhancing mural nodule	31 (62%)	16 (53.33%)	0.3
MPD >10 mm	10 (20.41%)	2 (7.14%)	0.11
Worrisome features *n* (%)			
Cyst size >3 cm	20 (38.46%)	6 (20.69%)	0.08
Pancreatitis	15 (29.41%)	7 (23.33%)	0.37
Enhancing cyst wall	16 (31.37%)	7 (25%)	0.37
MPD 5–10 mm	22 (44.9%)	16 (57.14%)	0.21
Abrupt change in calibre of pancreatic duct with distal pancreatic atrophy	24 (48.98%)	15 (53.57%)	0.44
CA19.9, median (IQR)	8.5 (2–39)	17.5 (3–107)	0.24
CEA, median (IQR)	1 (0.4–3)	2 (1–4.5)	0.27
Inflammatory biomarkers			
Neutrophils, median (IQR)	3.7 (2.9–4.7)	3.55 (2.9–5.3)	0.73
Lymphocytes, median (IQR)	1.5 (1.26–2.1)	1.5 (1.2–1.9)	0.62
Platelets, median (IQR)	223 (189–271)	241 (192–296)	0.48
CRP, median (IQR)	3 (2.9–5)	3 (2–4)	0.77
Albumin, median (IQR)	4.2 (4–4.4)	4.2 (4.1–4.4)	0.93
PNI ≥ 42.05, *n*%	4 (7.55%)	4 (13.33%)	0.31
NLR ≥ 2.38, *n*%	26 (49.86%)	16 (53.33%)	0.44
PLR ≥ 185.5 *n* %	13 (24.53%)	13 (43.33%)	0.06

## Data Availability

The data presented in this study are available on request from the corresponding author.

## References

[1] Bosman F.T., Carneiro F., Hruban R.H., Theise N.D. (2010). WHO Classification of Tumors of the Digestive System.

[2] Tanaka M., Fernández-del Castillo C., Kamisawa T., Jang J.Y., Levy P., Ohtsuka T., Salvia R., Shimizu Y., Tada M., Wolfgang C.L. (2017). Revisions of International Consensus Fukuoka Guidelines for the Management of IPMN of the Pancreas. Pancreatology.

[3] Kaimakliotis P., Riff B., Pourmand K., Chandrasekhara V., Furth E.E., Siegelman E.S., Drebin J., Vollmer C.M., Kochman M.L., Ginsberg G.G. (2015). Sendai and Fukuoka Consensus Guidelines Identify Advanced Neoplasia in Patients with Suspected Mucinous Cystic Neoplasms of the Pancreas. Clin. Gastroenterol. Hepatol..

[4] Kim J.R., Jang J.-Y., Kang M.J., Park T., Lee S.Y., Jung W., Chang J., Shin Y., Han Y., Kim S.-W. (2015). Clinical Implication of Serum Carcinoembryonic Antigen and Carbohydrate Antigen 19-9 for the Prediction of Malignancy in Intraductal Papillary Mucinous Neoplasm of Pancreas. J. Hepato-Biliary-Pancreat. Sci..

[5] Saito H., Kono Y., Murakami Y., Shishido Y., Kuroda H., Matsunaga T., Fukumoto Y., Osaki T., Ashida K., Fujiwara Y. (2018). Prognostic Significance of the Preoperative Ratio of C-Reactive Protein to Albumin and Neutrophil–Lymphocyte Ratio in Gastric Cancer Patients. World J. Surg..

[6] Gao J., Agizamhan S., Zhao X., Jiang B., Qin H., Chen M., Guo H. (2019). Preoperative C-Reactive Protein/Albumin Ratio Predicts Outcome of Surgical Papillary Renal Cell Carcinoma. Future Oncol..

[7] Xiang Z., Hu T., Wang Y., Wang H., Xu L., Cui N. (2020). Neutrophil–Lymphocyte Ratio (NLR) Was Associated with Prognosis and Immunomodulatory in Patients with Pancreatic Ductal Adenocarcinoma (PDAC). Biosci. Rep..

[8] Alagappan M., Pollom E.L., von Eyben R., Kozak M.M., Aggarwal S., Poultsides G.A., Koong A.C., Chang D.T. (2018). Albumin and Neutrophil-Lymphocyte Ratio (NLR) Predict Survival in Patients with Pancreatic Adenocarcinoma Treated With SBRT. Am. J. Clin. Oncol..

[9] Shen Y., Wang H., Li W., Chen J. (2019). Prognostic Significance of the CRP/Alb and Neutrophil to Lymphocyte Ratios in Hepatocellular Carcinoma Patients Undergoing TACE and RFA. J. Clin. Lab. Anal..

[10] Gemenetzis G., Bagante F., Griffin J.F., Rezaee N., Javed A.A., Manos L.L., Lennon A.M., Wood L.D., Hruban R.H., Zheng L. (2017). Neutrophil-to-Lymphocyte Ratio Is a Predictive Marker for Invasive Malignancy in Intraductal Papillary Mucinous Neoplasms of the Pancreas. Ann. Surg..

[11] Hata T., Mizuma M., Motoi F., Ishida M., Morikawa T., Nakagawa K., Hayashi H., Kanno A., Masamune A., Kamei T. (2020). An Integrated Analysis of Host- and Tumor-Derived Markers for Predicting High-Grade Dysplasia and Associated Invasive Carcinoma of Intraductal Papillary Mucinous Neoplasms of the Pancreas. Surg. Today.

[12] Li J.A., Han X., Fang Y., Zhang L., Lou W.H., Xu X.F., Wu W.C., Kuang T.T., Wang D.S., Rong Y.F. (2019). The value of preoperative CA19-9 combined with platelet-to-lymphocyte ratio in predicting invasive malignancy in intraductal papillary mucinous neoplasms. Zhonghua Wai Ke Za Zhi.

[13] Basturk O., Hong S.-M., Wood L.D., Adsay N.V., Albores-Saavedra J., Biankin A.V., Brosens L.A.A., Fukushima N., Goggins M., Hruban R.H. (2015). A Revised Classification System and Recommendations From the Baltimore Consensus Meeting for Neoplastic Precursor Lesions in the Pancreas. Am. J. Surg. Pathol..

[14] Tanaka M., Chari S., Adsay V., Carlos Castillo F.-D., Falconi M., Shimizu M., Yamaguchi K., Yamao K., Matsuno S. (2006). International Consensus Guidelines for Management of Intraductal Papillary Mucinous Neoplasms and Mucinous Cystic Neoplasms of the Pancreas. Pancreatology.

[15] Laird B.J., Kaasa S., McMillan D.C., Fallon M.T., Hjermstad M.J., Fayers P., Klepstad P. (2013). Prognostic Factors in Patients with Advanced Cancer: A Comparison of Clinicopathological Factors and the Development of an Inflammation-Based Prognostic System. Clin. Cancer Res..

[16] Tanaka M., Fernández-del Castillo C., Adsay V., Chari S., Falconi M., Jang J.-Y., Kimura W., Levy P., Pitman M.B., Schmidt C.M. (2012). International Consensus Guidelines 2012 for the Management of IPMN and MCN of the Pancreas. Pancreatology.

[17] Kwon W., Han Y., Byun Y., Kang J.S., Choi Y.J., Kim H., Jang J.-Y. (2020). Predictive Features of Malignancy in Branch Duct Type Intraductal Papillary Mucinous Neoplasm of the Pancreas: A Meta-Analysis. Cancers.

[18] Goh B.K.P., Tan D.M.Y., Chan C.-Y., Lee S.-Y., Lee V.T.W., Thng C.-H., Low A.S.C., Tai D.W.M., Cheow P.-C., Chow P.K.H. (2015). Are Preoperative Blood Neutrophil-to-Lymphocyte and Platelet-to-Lymphocyte Ratios Useful in Predicting Malignancy in Surgically-Treated Mucin-Producing Pancreatic Cystic Neoplasms?: NLR and PLR in Pancreatic Cysts. J. Surg. Oncol..

[19] Arima K., Okabe H., Hashimoto D., Chikamoto A., Kuroki H., Taki K., Kaida T., Higashi T., Nitta H., Komohara Y. (2015). The Neutrophil-to-Lymphocyte Ratio Predicts Malignant Potential in Intraductal Papillary Mucinous Neoplasms. J. Gastrointest. Surg..

[20] Hamada S., Masamune A., Shimosegawa T. (2014). Inflammation and Pancreatic Cancer: Disease Promoter and New Therapeutic Target. J. Gastroenterol..

[21] Sadot E., Basturk O., Klimstra D.S., Gönen M., Lokshin A., Do R.K.G., D’Angelica M.I., DeMatteo R.P., Kingham T.P., Jarnagin W.R. (2015). Tumor-Associated Neutrophils and Malignant Progression in Intraductal Papillary Mucinous Neoplasms: An Opportunity for Identification of High-Risk Disease. Ann. Surg..

[22] Inman K.S. (2014). Complex Role for the Immune System in Initiation and Progression of Pancreatic Cancer. World J. Gastroenterol..

[23] Arima K., Okabe H., Hashimoto D., Chikamoto A., Tsuji A., Yamamura K., Kitano Y., Inoue R., Kaida T., Higashi T. (2016). The Diagnostic Role of the Neutrophil-to-Lymphocyte Ratio in Predicting Pancreatic Ductal Adenocarcinoma in Patients with Pancreatic Diseases. Int. J. Clin. Oncol..

[24] McIntyre C.A., Pulvirenti A., Lawrence S.A., Seier K., Gonen M., Balachandran V.P., Kingham T.P., D’Angelica M.I., Drebin J.A., Jarnagin W.R. (2019). Neutrophil-to-Lymphocyte Ratio as a Predictor of Invasive Carcinoma in Patients with Intraductal Papillary Mucinous Neoplasms of the Pancreas. Pancreas.

[25] Ohno R., Kawamoto R., Kanamoto M., Watanabe J., Fujii M., Ohtani H., Harada M., Kumagi T., Kawasaki H. (2019). Neutrophil to Lymphocyte Ratio Is a Predictive Factor of Malignant Potential for Intraductal Papillary Mucinous Neoplasms of the Pancreas. Biomark. Insights.

[26] Hata T., Mizuma M., Motoi F., Ishida M., Morikawa T., Takadate T., Nakagawa K., Hayashi H., Kanno A., Masamune A. (2019). Diagnostic and Prognostic Impact of Neutrophil-to-Lymphocyte Ratio for Intraductal Papillary Mucinous Neoplasms of the Pancreas With High-Grade Dysplasia and Associated Invasive Carcinoma. Pancreas.

[27] Goh B.K.P., Teo J.-Y., Allen J.C., Tan D.M.Y., Chan C.-Y., Lee S.-Y., Tai D.W.M., Thng C.-H., Cheow P.-C., Chow P.K.H. (2016). Preoperative Platelet-to-Lymphocyte Ratio Improves the Performance of the International Consensus Guidelines in Predicting Malignant Pancreatic Cystic Neoplasms. Pancreatology.

[28] Bhatti I., Peacock O., Lloyd G., Larvin M., Hall R.I. (2010). Preoperative Hematologic Markers as Independent Predictors of Prognosis in Resected Pancreatic Ductal Adenocarcinoma: Neutrophil-Lymphocyte versus Platelet-Lymphocyte Ratio. Am. J. Surg..

[29] Ahmad J., Grimes N., Farid S., Morris-Stiff G. (2014). Inflammatory Response Related Scoring Systems in Assessing the Prognosis of Patients with Pancreatic Ductal Adenocarcinoma: A Systematic Review. Hepatobiliary Pancreat. Dis. Int..

[30] Stotz M., Gerger A., Eisner F., Szkandera J., Loibner H., Ress A.L., Kornprat P., A Zoughbi W., Seggewies F.S., Lackner C. (2013). Increased Neutrophil-Lymphocyte Ratio Is a Poor Prognostic Factor in Patients with Primary Operable and Inoperable Pancreatic Cancer. Br. J. Cancer.

[31] Shusterman M., Jou E., Kaubisch A., Chuy J.W., Rajdev L., Aparo S., Tang J., Ohri N., Negassa A., Goel S. (2020). The Neutrophil-to-Lymphocyte Ratio Is a Prognostic Biomarker in An Ethnically Diverse Patient Population with Advanced Pancreatic Cancer. J. Gastrointest. Cancer.

[32] Asari S., Matsumoto I., Toyama H., Shinzeki M., Goto T., Ishida J., Ajiki T., Fukumoto T., Ku Y. (2016). Preoperative Independent Prognostic Factors in Patients with Borderline Resectable Pancreatic Ductal Adenocarcinoma Following Curative Resection: The Neutrophil-Lymphocyte and Platelet-Lymphocyte Ratios. Surg. Today.

[33] Zhou Y., Wei Q., Fan J., Cheng S., Ding W., Hua Z. (2018). Prognostic Role of the Neutrophil-to-Lymphocyte Ratio in Pancreatic Cancer: A Meta-Analysis Containing 8252 Patients. Clin. Chim. Acta.

[34] Haruki K., Shiba H., Shirai Y., Horiuchi T., Iwase R., Fujiwara Y., Furukawa K., Misawa T., Yanaga K. (2016). The C-Reactive Protein to Albumin Ratio Predicts Long-Term Outcomes in Patients with Pancreatic Cancer After Pancreatic Resection. World J. Surg..

[35] Liu Z., Jin K., Guo M., Long J., Liu L., Liu C., Xu J., Ni Q., Luo G., Yu X. (2017). Prognostic Value of the CRP/Alb Ratio, a Novel Inflammation-Based Score in Pancreatic Cancer. Ann. Surg. Oncol..

[36] Kinoshita A., Onoda H., Imai N., Iwaku A., Oishi M., Tanaka K., Fushiya N., Koike K., Nishino H., Matsushima M. (2015). The C-Reactive Protein/Albumin Ratio, a Novel Inflammation-Based Prognostic Score, Predicts Outcomes in Patients with Hepatocellular Carcinoma. Ann. Surg. Oncol..

[37] Proctor M.J., Morrison D.S., Talwar D., Balmer S.M., O’Reilly D.S.J., Foulis A.K., Horgan P.G., McMillan D.C. (2011). An Inflammation-Based Prognostic Score (MGPS) Predicts Cancer Survival Independent of Tumour Site: A Glasgow Inflammation Outcome Study. Br. J. Cancer.

[38] Wong V.K.H., Malik H.Z., Hamady Z.Z.R., Al-Mukhtar A., Gomez D., Prasad K.R., Toogood G.J., Lodge J.P.A. (2007). C-Reactive Protein as a Predictor of Prognosis Following Curative Resection for Colorectal Liver Metastases. Br. J. Cancer.

[39] Yang J., Wezeman M., Zhang X., Lin P., Wang M., Qian J., Wan B., Kwak L.W., Yu L., Yi Q. (2007). Human C-Reactive Protein Binds Activating Fcgamma Receptors and Protects Myeloma Tumor Cells from Apoptosis. Cancer Cell.

[40] Kanda M., Fujii T., Kodera Y., Nagai S., Takeda S., Nakao A. (2011). Nutritional Predictors of Postoperative Outcome in Pancreatic Cancer. Br. J. Surg..

[41] Okumura S., Kaido T., Hamaguchi Y., Fujimoto Y., Masui T., Mizumoto M., Hammad A., Mori A., Takaori K., Uemoto S. (2015). Impact of Preoperative Quality as Well as Quantity of Skeletal Muscle on Survival after Resection of Pancreatic Cancer. Surgery.

